# Genetic Polymorphism and serum levels of Insulin like Growth Factor-1 (Igf-1) in patients of rheumatoid arthritis

**DOI:** 10.12669/pjms.39.3.6561

**Published:** 2023

**Authors:** Iram Fayyaz, Saba Khaliq, Farhat Bano, Haiba Kaul

**Affiliations:** 1 Iram Fayyaz, MBBS, DCH, M.Phil. Department of Biochemistry, University of Health Sciences, Lahore, Pakistan; 2 Saba Khaliq, PhD Department of Physiology, University of Health Sciences, Lahore, Pakistan; 3 Farhat Bano, PhD Department of Biochemistry, University of Health Sciences, Lahore, Pakistan; 4 Haiba Kaul, PhD Department of Genetics, University of Veterinary and Animal Sciences, Lahore, Pakistan

**Keywords:** Insulin like growth factor-1, Genetic polymorphism, Rheumatoid arthritis, Severity of disease

## Abstract

**Objectives:**

Insulin like growth factor-1(IGF-1), is a modulator of immunity and inflammation, it promotes the anabolic role of growth hormone (GH) on bone and skeletal tissue. Genetic polymorphism in IGF-1 gene is reported to affect the transcriptional efficiency affecting its serum level. In this study we aim: 1) To study the presence of 192bp polymorphism of IGF-1 gene in patients of rheumatoid arthritis (RA), 2) To study the association of 192 bp polymorphism of IGF-1 gene with serum IGF-1 levels and disease severity in patients of RA.

**Methods:**

A cross-sectional study was carried out at University of Health Sciences (UHS), Lahore. Diagnosed RA cases who fulfilled the American College of Rheumatology (ACR) criteria were recruited from Fatima Memorial Hospital (FMH) and Behbud Rheumatology Clinics, Lahore during 2018–2019. Serum IGF-1 levels were determined by ELISA in blood samples of 200 RA patients and 200 healthy individuals. DNA was extracted and genetic polymorphism was determined.

**Results:**

The serum IGF-1 level in RA group was significantly lower compared to healthy group. Our study shows presence of 192bp allele of IGF-1in 77% of the studied population. Carriers of 192bp allele of IGF-1 had a significantly higher serum level of IGF-1 as compared to non-carriers in the RA patients. Rheumatoid factor (RF) positive patients had a higher number of 192bp carriers in comparison to RF negative patients. Significant difference was also seen in severity of disease between carrier and non-carriers of 192bp allele with the disease being more severe in male carriers.

**Conclusions:**

There is an association of IGF-1gene polymorphism with variation in serum IGF-1 levels and severity of RA.

## INTRODUCTION

Rheumatoid arthritis (RA) is a chronic autoimmune disease, which causes painful inflammation of joints which leads to chronic disability. It typically affects the joints of hands and feet i.e., elbows, wrists, ankles and knee which gradually compromises the ability to perform routine daily activities.^1^ There is dearth of data related to RA prevalence in developing countries like Pakistan. Tertiary care hospitals in Pakistan proposed that the prevalence of RA in the southern versus northern regions of the country is 0.142%-5.5% respectively.^2^ In a systematic review, Naqvi et al, reported that very few studies on RA patients have been conducted in Pakistan over the last decade, and in the studies which were conducted the patients were mostly poor females; uneducated and unemployed.^3^ A cross-sectional study conducted in Rheumatology Department, Fatima Memorial Hospital, Lahore, Pakistan, from May, 2018 to July, 2018 reported that delay in disease diagnosis and therapeutic intervention ultimately led to high disease activity and poor functional outcome in RA patients.^4^ It is indeed the need of the hour to address these factors to improve outcome of RA in Pakistani public.

RA is a systemic, chronic, inflammatory disease of unknown etiology. It is characterized by immune activation, leucocyte infiltration and synovial inflammation, leading to eventual joint pain and swelling.^5^ The future of RA drug treatment lies in identifying the elevated pro-inflammatory elements in both the serum and synovial fluid of individual RA patients prior to starting drug therapy thus producing “personalized molecular medicine”.^6^

There are ongoing studies regarding involvement of genetic polymorphism in RA in Pakistan as well. Recently, a study was carried out in Karachi to find out the association of SNPs (*rs708035* and *rs3844283)* with *IRAK2* gene which may affect increase and decrease of NFκB levels either by promoting or reducing different proteins affecting bone health that might be responsible for progression of RA.^7^ Another recent study on RA in Pakistan focused on role of auto-antibody against mutated citrullinated vimentin (Anti-MCV) which was shown to have the same diagnostic value as ACPA in Pakistani RA patients but is a better predictor of disease progression.^8^ In a recent study on RA genetics and IGF-IR, the homozygous AA genotype form; +3179G/A IGF-IR was found to be linked with increased likelihood of developing RA.^9^ An important variant in the promoter of IGF-1 gene has been previously shown to increase RA susceptibility, associated with low IGF-I serum levels and higher disease activity score particularly in male individuals who were non-carriers of the wild type 192-bp allele.^10^ IGF-1 production occurs throughout life reaching highest levels in adolescence and falling to the lowest in infancy and old age.^11^ Identification of further such genetic factors in our setting will help identify methods of early diagnosis and treatment to prevent chronic disability as seen in RA in Pakistani patients. It would also aid in understanding the complexities of this disabling disease, by providing necessary base line data for the Pakistani population.

## METHODS

The current study was conducted and approved in accordance with the rules and regulations stipulated by Ethics Review Committee, University of Health Sciences, Lahore, Pakistan (No. UHS/REG-18/ERC/184). All RA diagnosed cases and healthy individuals, included in the study were recruited during 2018–2019 after taking informed written consent from all participants. The RA patients were selected on the criteria established by American College of Rheumatology (ACR). Two hundred RA patients attending the rheumatology clinics at Behbud center and Fatima Memorial hospital, Lahore, Pakistan were recruited. The relevant clinical data was collected, i.e. swollen joint count (SJC), tender joint count (TJC), general health (GH), and erythrocyte sedimentation rate (ESR). This was used to find the “disease activity score of 28 joints (DAS28)” for all RA patients using the following formula:

“0.56 x (TJC28) +0.28 x (SJC28) +0.70 x

ln(ESR) + 0.014 x (GH)”^12^

The ratings of patients’ general health were given by them on a “100-mm visual analogue scale (VAS)”. Two hundred apparently healthy subjects with no history of RA were recruited as controls.

Ten ml venous blood was obtained by sterile venipuncture. Five ml blood was transferred into vacutainers containing EDTA and was kept at -20°C till DNA extraction whereas 5 ml blood was used for serum isolation which was stored for serum biochemistry. Serum was obtained by centrifugation (3000 rpm for 10 minutes) and stored at -80°C**.**

### Genotyping of IGF-1 and Immunoassay:

“Favor Prep Blood Genomic DNA Extraction Kit (Biotech Corp, Taiwan China)” was used for extracting DNA. Polymerase chain reaction (PCR) was employed to amplify DNA using specific primers (Macrogen Inc Korea) designed against the polymorphic cytosine–adenine repeat 1kb upstream of the human IGF-1 gene, i.e., sense: 5’-ACCACTCTGGGAGAAGGGTA-3, antisense: 5-GCTAGCCAGCTGGTGTTATT-3’. PCR amplification reactions were carried out in a 25μL reaction mixture which contained, 1μl (50 ng) of genomic DNA, 0.5 μl of each primer (5pM), 12.5 μl of 2x PCR master mix and 10.5 μl double distilled water. Amplification was performed on BioRad thermal cycler (iCycler, BioRad USA). The cycling conditions for the PCR were a cycle of initial denaturation at 94ºC for five minutes, followed by 35 cycles with denaturation for one minute at 94ºC, annealing at 58ºC for 60 seconds, extension at 72ºC for 60 sec, and with a final extension for 10 minutes. at 72ºC. The amplicons were run for 45 minutes on 3% agarose gel. The PCR product was checked under ultraviolet gel documentation system (Bio-Rad USA) and results were recorded.

IGF-1 levels in serum samples of both RA group and healthy group was measured by Enzyme Immunoassay kit (Bioassay technology Laboratory Shanghai China) following manufacturer’s instructions on automated ELISA CODA (BioRad, USA).

### Statistical analysis:

Data was analyzed using the version 22 of SPSS (Statistical Package for Social Sciences). Frequencies and percentages are given for categorical variables. Data distribution was checked by Shapiro-Wilk’s statistics. Mean+SD (standard deviation) was given for normally distributed quantitative variables and median with IQR (Interquartile range) was given for non-normally distributed quantitative variables. Student’s t test and Mann-Whitney U test were applied to compare normally or non-normally distributed quantitative variables respectively between two groups. Genotype frequencies between the two groups were compared by Chi square/Fisher’s exact test. A p value ≤0.05 was considered significant.

## RESULTS

This study aimed to study the serum levels of IGF-1 in both RF +ive and RF –ive RA patients as well as healthy controls, and to observe the frequency with which 192-bp allele of IGF-1 appeared in the study groups, its relationship to serum IGF-1 levels and also if it bore any relation to disease severity. In RA patient group, 142(71%) were carriers of 192bp allele and 58(29%) were non-carrier of 192bp allele. In healthy group, 165(82.5%) were carriers of 192bp allele and 35(17.5%) were non-carriers. There was a significant difference observed in the frequency of 192bp allele in groups (*p*=0.006) (Table-I). On basis of gender stratification, it was observed that out of male RA patients 20(60.6%) were carriers and 13(39.3%) were non-carriers of 192bp allele (*p*=0.12). Out of female RA patients, 123(73.65%) were carriers and 44(26.34%) were non-carriers of 192bp allele. The frequency of 192bp allele in healthy males was 34(85%) whereas 6(15 %) males were non-carriers of 192bp allele. In female controls the frequency of 192bp allele carriers was 131(81.87%), and 29(18.12%) were non-carriers (*p*=0.64).

**Table-I:** Comparison of IGF-1 192 bp polymorphism between the RA patients and healthy group.

Genotype	RA Patients Group (n=200)	Healthy Group (n=200)	Chi square	p-value	Confidence Interval
Non Carrier of 192 bp	58(29)	35(17.5)	7.41	0.006	1.2-3.1
Carrier of 192 bp	142(71)	165(82.5)			

Values given as n(%). A “Chi Square” test was utilized to calculate the p-value and “Confidence Interval” (CI). A “ p” of<0.05 was considered significant.

Out of RF +ive patients, 112(76.55%) were carriers and 34(23.44%) were non-carriers of 192bp allele. Out of RF –ive patients, 31 (56.6%) were carriers and 23(43.4%) were non carriers of 192bp allele of IGF-1. A Significant difference in 192 bp carrier status in RF +ive and RF-ive patients was observed (*p*=0.007) (Table-II).

**Table-II:** Comparison of IGF-1, 192 bp carrier state in RA patients and healthy group according to RA factor.

Genotype	RA Patient Group (n=200)		Chi square p-value CI	Healthy Group (n=200)		Chi square p-value CI
	
	RF (+ve)	RF (–ve)		RF(+ve)	RF (–ve)	
Non Carrier of 192 bp	34(23)	23(43)	7.2 0.007 0.2-0.7	5(45)	30(16)	6.3 0.01 1.2-15.4
Carrier of 192 bp	112(77)	31(57)		6(55)	159(84)	

Values given as n(%). A “Chi Square” test was utilized to calculate the p-value and “Confidence Interval” (CI). A “p” of<0.05 was considered significant.

After gender stratification, severity of disease was analyzed in carriers and non-carriers of 192bp allele (Table-III), male RA patients who were carriers of 192 bp showed more cases of severe disease based on DAS 28 score (*p*=0.045). Severe disease although seen more in female carriers but the difference was not significant (*p*=0.167).

**Table-III:** Comparison of DAS 28 score and IGF-1 polymorphism in male and female RA patients.

Gender	DAS Score	Non-Carrier of 192 bp n(%)	Carrier of 192 bp n(%)	Fisher’s exact p-value CI
Male (n=33)	Remission	5(38)	5(25)	5.030 0.045 (0.163-0.177)
	Low disease activity	7(54)	6(30)	
	Moderate disease activity	1(8)	5(25)	
	High disease activity	0(0)	4(20)	
Female (n=167)	Remission	6(13)	33(27)	3.435 0.167 (0.314-0.332)
	Low disease activity	13(29)	30(25)	
	Moderate disease activity	23(51)	51(42)	
	High disease activity	3(7)	8(6)	

Values given as n(%). A “Fisher,s exact ” test was utilized to calculate the p-value and “Confidence Interval” (CI). A “p” of<0.05 was considered significant.

**Fig.1:**
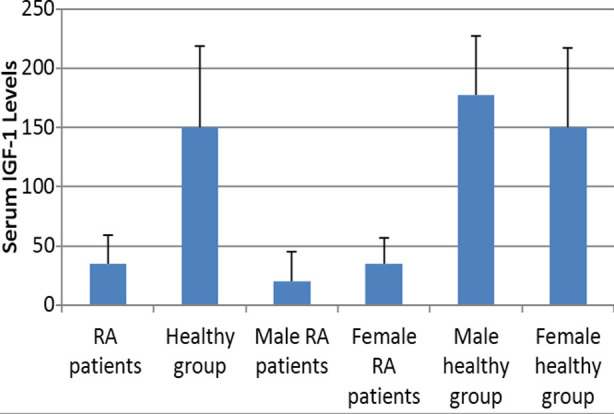
Comparison of Serum IGF-1 levels in Patients and Controls.

The serum IGF-1 levels were non-normally distributed in the population. There was a significant difference in the median±IQR of IGF-1 serum levels in RA patients [35(24)] as compared to healthy group [150(69.52)] (*p*<0.001). Serum levels of IGF-1 were significantly higher in female RA cases and male healthy controls. In the patient group, males had an IGF-1 level of 20(25.1) while females had a level of 35(22.8), (*p*=0.006). In the healthy group, males had a level of IGF-1 at 177.5(50) while females had a reading of 150(68.75) (*p*=0.017) (Fig.1).

**Fig.2:**
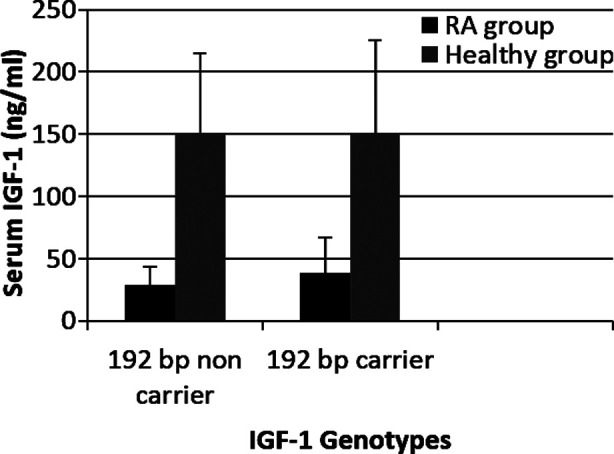
Comparison of Serum IGF-1 levels in IGF-1 192 bp carriers and non-carriers in RA patients and healthy group.

Median (IQR) of serum IGF1in RA patients was 39(27.86%) in carriers of 192 bp allele and 29 (15%) in the non-carriers (*p*=0.001). In healthy group the serum level was 150(75.50%) for carriers and 150(65%) for non-carriers of 192 bp allele (*p*=0.08). Significant difference in serum IGF1 levels between carriers and non-carriers was seen in the RA patient group (Fig.2).

## DISCUSSION

In our study there is a significant difference in serum IGF-1 levels between the two study groups, with lower serum levels found in RA patients’ group. Disturbances in IGF1/IGF1R signaling are notable for RA pathology. It contributes to joint inflammation and affects homeostasis in chondrocytes, leukocytes and synovial fibroblasts.^13^ Similar results were seen in a previously conducted meta-analysis which showed that patients with RA have decreased levels of IGF-1 in circulation as compared to healthy individuals, especially in the Asian and European communities; however, result was not significant in American study group.^14^ In another meta-analysis decreased levels of IGF-1 are seen to be linked with RA, moreover race and age were determined as the influencing factors.^15^ There are also studies which reported that there was no difference in the levels between the two groups.^16^ Baker et al., reported that IGF-1 levels were progressively lower with more severe and prolonged RA and there was an association between IGF-1 levels, disease activity, disease duration, joint destruction and disability. This observation helps support the hypothesis that RA patients have a dysregulation in GH/IGF-1 signaling pathway most likely because of an acquired resistance to GH.^17^ In another study, on correlation between DAS28 and IGF-1 in RA patients , IGF-1 levels showed a significant difference between the high-disease-activity and low-disease-activity groups.^18^ Interestingly physical inactivity, a condition acquired in long-lasting RA is frequently associated with lower serum IGF-1.^19^ Changing lifestyle with increased exercise to improve serum IGF-1 levels could be an alternative therapy for RA patients.^20^

Another area of interest is the contribution of IGF-1 & insulin like growth factor binding protein (IGFBP) axes in RA pathogenesis. This has been examined in many studies but controversial results have been obtained. There were studies which reported high IGFBP and IGF-Ι levels in both synovial fluid (SF) and serum of RA patients when compared to healthy group.^21^ There are also studies on the contrary reporting similar or reduced IGF-I and IGFBP levels in RA patients compared to controls.^22^ In RA, IGF-1 protein expression is deranged, which results in decreased blood levels of IGF-1 acting on the regulatory T-cells (Treg) and decreased suppression of inflammation. IGF-1 based therapy is thus proposed for treating such chronic autoimmune diseases as it has a role in modulating the IGF-1/IGF-IR (IGF-1 receptor) signaling pathway and thus modulating immune response and Treg cell proliferation.^23^

The present study is an initial exploration of the possible genetic impact of 192bp polymorphism and serum levels of IGF-1 as a disease predisposing factor for RA in Pakistani population. Our study showed presence of 192 bp allele of IGF-1 in 77% population. A population-based study reports presence of 192-bp allele in almost 88% of the general population.^24^ Another study observed that only a minor 3% of all the study participants displayed the absence of 192-bp allele of IGF-1.^10^

Our study showed a significant difference in serum IGF-1 levels between carriers and non-carriers of 192bp allele in RA patients, with higher serum level seen in carriers. In a previous study, it was seen that individuals carrying the 192bp allele had a significantly higher level of IGF-1 compared with non-carriers, this was in accordance with our findings indicating that 192bp allele effects the serum level of IGF-1.^25^ Another population-based study suggests that higher the number of 192bp alleles carried, higher the serum IGF-1 level.^24^ In one study, it is reported that male patients who were non-carriers of 192bp allele had lower serum IGF-1 levels but it was not statistically significant.^10^

In our study, a significant difference in 192bp carrier status was seen between RF+ive and RF-ive patients. In our study, a significant difference was also observed in severity of disease between carriers and non-carriers of 192bp allele after applying gender stratification with the disease being more severe in male carriers of 192 bp allele. Contrary to our results, a previous study reports more severe disease was seen in non-carriers of 192 bp allele. 10

### Limitation of the study:

The limitation of the present study is the small sample size. As this is a small scale study, our assumptions need to be confirmed in further studies. Furthermore, it’s difficult to establish fully, the pathogenic role of the SNPs by evaluating the importance of single gene effects. However, the strength of the study is that there is lack of similar data from Pakistan in the literature. A larger sample size and multicentric study should be designed with focus on more genetic analysis as ours is a pilot study providing the foundation for future studies.

## CONCLUSIONS

To conclude, our findings suggest that there is variation in serum IGF-1 levels due to presence or absence of 192bp allele of IGF-1. Carriers of 192bp allele have higher serum levels of IGF-1 in our study as well as various previous studies. As IGF-1 has a protective growth modulatory role, it’s deficiency could also have a role in the pathogenesis of RA. Moreover, there is an association of severity of RA with IGF-1 gene polymorphism, however, results are contradictory as our study showed more severe disease in carriers while a previous study showed greater severity of RA in non carriers of 192 bp allele. Clearly further research is required to elucidate the correlation of IGF-1 and its genetic variants with the RA disease activity.

### Authors’ Contribution:

**IF:** Sample collection, experimental laboratory work, data entry and manuscript writing.

**SK:** Supervised research, Statistical analysis and interpretation of data, Review and final approval of the manuscript.

**FB:** Review of the statistical analysis.

**HK:** Study design, supervised research, review and final approval of the manuscript.

All the authors are responsible and accountable for the accuracy or integrity of the work.
